# Semantic Segmentation of Remote Sensing Images Depicting Environmental Hazards in High-Speed Rail Network Based on Large-Model Pre-Classification

**DOI:** 10.3390/s24061876

**Published:** 2024-03-14

**Authors:** Qi Dong, Xiaomei Chen, Lili Jiang, Lin Wang, Jiachong Chen, Ying Zhao

**Affiliations:** 1 School of Optics and Photonics, Beijing Institute of Technology, Beijing 100081, China; 3220210451@bit.edu.cn (Q.D.); 3220210489@bit.edu.cn (L.W.); 3120220537@bit.edu.cn (J.C.); 3220220567@bit.edu.cn (Y.Z.); 2 Key Laboratory of Photoelectronic Imaging Technology and System, Ministry of Education, Beijing Institute of Technology, Beijing 100081, China; 3 China Academy of Railway Sciences Corporation Limited, Beijing 100081, China; jianglili@rails.cn

**Keywords:** remote sensing images, high-speed railway, color-coated steel sheet roof buildings, segment anything model

## Abstract

With the rapid development of China’s railways, ensuring the safety of the operating environment of high-speed railways faces daunting challenges. In response to safety hazards posed by light and heavy floating objects during the operation of trains, we propose a dual-branch semantic segmentation network with the fusion of large models (SAMUnet). The encoder part of this network uses a dual-branch structure, in which the backbone branch uses a residual network for feature extraction and the large-model branch leverages the results of feature extraction generated by the segment anything model (SAM). Moreover, a decoding attention module is fused with the results of prediction of the SAM in the decoder part to enhance the performance of the network. We conducted experiments on the Inria Aerial Image Labeling (IAIL), Massachusetts, and high-speed railway hazards datasets to verify the effectiveness and applicability of the proposed SAMUnet network in comparison with commonly used semantic segmentation networks. The results demonstrated its superiority in terms of both the accuracies of segmentation and feature extraction. It was able to precisely extract hazards in the environment of high-speed railways to significantly improve the accuracy of semantic segmentation.

## 1. Introduction

**Figure 1:**
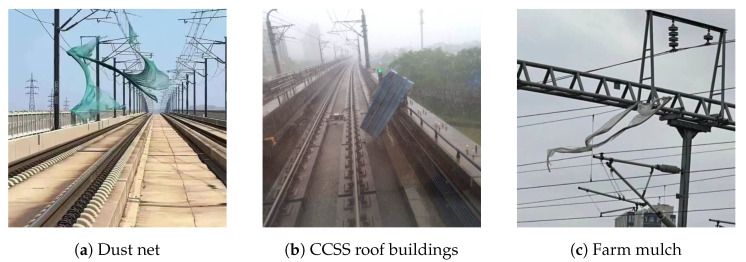
Accidents caused by high-speed rail hazards.

With the rapid development of high-speed railway networks in recent years, high-speed trains have become an indispensable means of transportation for the public [1]. With progress in the deployment of the new generation of high-speed electric multiple unit (EMU) trains that can travel at 400 km/h [2], it has become crucial to ensure the safety of the operating environment of high-speed trains. The periodic inspection and monitoring of hazards in the environment of high-speed railways are necessary for ensuring the safety of their operating environment [3]. There are two types of major safety hazards in the vicinity of high-speed railways: light and heavy floating objects. Commonly encountered light floating objects include farm mulch and dust nets [4], as shown in Figure 1, while heavy floating objects mostly include color-coated steel sheet (CCSS) roof buildings. These structures are often illegally constructed by using simple and unreliable methods and can be blown onto high-speed railway tracks in windy weather to cause accidents [5]. The regular inspection of railway tracks to identify such hazards is thus important to ensure the safety of operation of high-speed trains.

**Figure 2:**
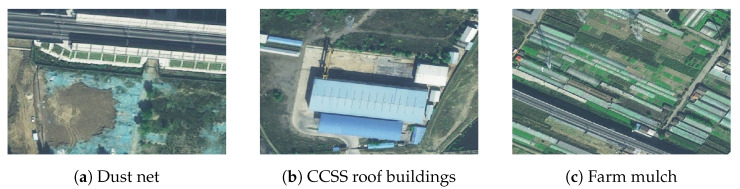
High-speed rail hazards.

However, the high-speed railway network covers a large area, parts of which are rarely visited by people. This makes manual inspection challenging. Due to its efficiency, high image resolution, wide coverage, and simple method of data acquisition, remote sensing technology has been used to monitor hazards in the environment of railway networks [6]. Currently used target detection technology based on remote sensing images primarily focuses on various targets, such as buildings [7,8], water resources [9,10], and farmland [11,12]. The study of railway hazards has emerged in recent years, and existing work has only been directed at the target of color-coated steel sheet roof buildings. Prevalent methods for extracting color-coated steel sheet roof buildings from remote sensing images can be classified into two categories: methods based on spectral indices, and those based on deep learning. In the context of methods based on spectral indices, Guo et al. [13] used Landsat remote sensing images to develop ’blue steel tile’-roofed buildings (BSTBs). They leveraged the characteristics of visible and near-infrared reflection of the target to obtain an accuracy of detection of 85%. Zhao et al. [14] used data from the Sentinel-2 satellite to construct the blue color-coated steel roofs (BCCSRs) index based on the blue, red, green, and short-wave infrared bands and reported an overall accuracy of 93%. In the context of deep learning-based techniques, Sun et al. [15] used image-related data from GF-2 and the DeepLabV3+ model of semantic segmentation to analyze the number and distribution of blue-colored steel plates in the Foshan region of China and obtained an accuracy of 92%. To address the issue of the complex background in remote sensing images, Li et al. [16] introduced the deformation-aware feature enhancement module (DFEM) and the feature alignment gated fusion module (FAGM) to the deep learning network. This led to improved accuracy in terms of extracting the colored steel roofs of houses from images. All of the above studies are aimed at a single target and cannot be adapted to the complex environment of the railway as shown in Figure 2.

Although the above studies have achieved excellent performance in terms of extracting color-coated steel sheet roof buildings from images, challenges persist in identifying hazards around high-speed railways. The spectral index-based approach relies on specific data from satellite remote sensing images, and the indices of their spectral features are significantly affected by the seasons and the climate. Moreover, multi-spectral remote sensing images have a low resolution that makes them unsuitable for use in high-resolution and complex remote sensing environments. Furthermore, a limited amount of research has been devoted to extracting railway hazards from remote sensing images, where this raises questions about the scope of applicability of the spectral features. On the contrary, the deep learning-based approach has unique advantages in terms of processing datasets of images and can adapt to data from different remote sensing satellites. However, most relevant studies have borrowed their design principles from network structures in other fields, because of which the capability of the corresponding methods to fit features of railway hazards in images has not been adequately verified.

Large models have recently been used for image segmentation to assist in the task of feature extraction. The segment anything model (SAM), released by Meta AI [17], can serve as a foundational model in the domain of image segmentation. This model is trained on a large number of unlabeled datasets and can be used for remote sensing without requiring additional training data [18,19]. This characteristic is advantageous for the identification of railway hazards around high-speed railways, as it can reduce the demand for labeled data. Hence, we consider using the SAM as a branch feature extraction module and leverage the capability of generalization of its pre-training dataset to enhance the performance of the network in terms of feature extraction. The ultimate goal is to improve the accuracy of extraction of railway hazards from images.

In response to the limited research on the extraction of railway hazards around high-speed railways from images and the fact that the existing research only focuses on color-coated steel roof buildings, which is a single target and cannot be adapted to the actual scenarios, we propose a dual-branch semantic segmentation network with the fusion of large models (SAMUnet) to achieve multi-target railway hazards segmentation in this study. Our approach combines a large model for semantic image segmentation with spectral features to construct a branch feature extraction module. Moreover, we design a multi-feature attention fusion module to efficiently integrate the features obtained by the network. This integration is designed to extract railway hazards around high-speed railways from remote sensing images with complex environmental backgrounds. The proposed method reduces reliance on traditional modules for feature extraction and enhances the precision of feature fusion by the model.

## 2. Architecture of SAMUnet

### 2.1. Feature Extraction Based on SAM

Remote sensing images contain a large number of details and complex spatial information due to improvements in their resolution. Current models of semantic segmentation struggle to segment complex datasets of images. Such issues as regional misjudgment and the loss of local information on hazardous targets are commonly encountered in images of high-speed railway lines with intricate spatial information. Some researchers have proposed a combination of traditional algorithms of image classification and deep learning models. They achieve this by adding additional branch structure inputs to reduce the data-related requirements and improve the accuracy of the model. However, traditional methods of threshold segmentation have limited accuracy, and their application to the environment of the railway is affected by various factors, such as the seasons and the weather, which make it challenging for them to effectively train the network model. The SAM image segmentation algorithm proposed by the Meta team offers a new approach. It relies on active learning and large-scale datasets to obtain zero-shot segmentation, which means that it can perform image segmentation without being specifically trained on a given dataset. The pre-training weights of the SAM are obtained from its SAM-1B dataset, which contains images of various locations and fields from all over the world. This dataset contributes to the high capability of generalization of the model and enables it to segment input images even in scenarios for which it has not been trained.

However, without specific guidance, the SAM tends to segment images into as many categories as possible such that this results in numerous categories of images without clear labels. A large number of segmented categories of images can lead to the fragmentation of their complex features in applications. Therefore, we use the SAM network for the unsupervised segmentation of remote sensing images. The mean values of the spectral bands of each segmented category are used as regional features to create feature maps, thus combining the capability of generalization of the large SAM with the spectral information of the original image.

**Figure 3:**
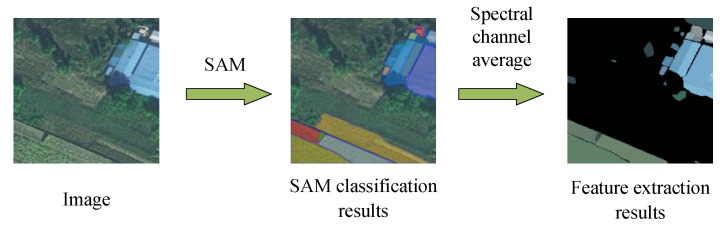
Schematic diagram of feature extraction by the SAM.

By considering safety hazards in the environment of high-speed railways, such as color-coated steel sheet roof buildings and sand control nets that have consistent internal spectral characteristics, we aim to minimize the fragmentation of the feature maps of the target. The process of extraction and its results are depicted in Figure 3. The original image is segmented by using the SAM to obtain regions of classification, and spectra of the original image are averaged within each region of classification to obtain a new feature map. The calculation is as follows:

In Algorithm  1, the input is Image and SAM large model, and the output is a Feature Feature map, $Are_{nclasses}$ and nclasses represent the number of segmentation categories obtained by SAM segmentation, and the image of each class, avg represents the spectral mean operation, and sum represents the area feature map stitched together to obtain the final overall feature map.
**Algorithm 1** SAM Pre-classification**Input:** Image, SAM**Output:** feature    1:$Are_{nclasses},nclasses=SAM\left(image\right)$2:**for** i=0 to nclasses **do**3:   $Feature_{nclasses}=avg\left(Are_{nclasses}\right)$4:**end for**5:$Feature=sum(Feature_{nclasses})$6:**return** Feature

### 2.2. Semantic Image Segmentation Based on SAMUnet Network

**Figure 4:**
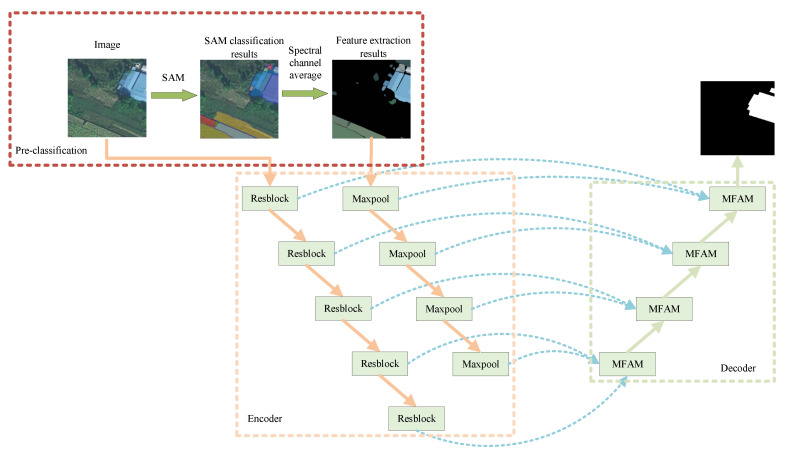
Structure of the SAMUnet model.

Research on the extraction of color-coated steel sheet roof buildings in the environment of high-speed railways based on remote sensing images is still in its early stages. Currently available techniques for extracting features from images of color-coated steel sheet roof buildings have limited applicability owing to their specific spatial and spectral resolutions, where this makes it difficult to apply them universally. We propose a dual-branch network for feature extraction based on a large model (SAMUnet). Figure 4 shows the framework of SAMUnet, which consists of an encoding and a decoding network. In the SAM pre-segmentation module, the SAM large model pre-classification results are combined with the original spectrum to obtain a new feature map as the subsequent network input. The encoding network is composed of modules for residual feature extraction (Resblock) and maximum pooling-based downsampling (Maxpool) and uses a dual-branch structure. The main branch uses a residual network (ResNet) to extract features from the input image and gradually downsamples them to reduce the scale of the feature map to learn abstract features from it. The auxiliary branch performs max pooling-based downsampling on the SAM map of spectral feature fusion. The decoding network contains a multi-feature attention fusion module (MAFM) that fuses spectral features with feature maps at the same scale. Skip connections are used to merge feature maps at the same scale to enable the network to capture large-scale contextual information and improve the accuracy of segmentation. Bilinear interpolation is used for upsampling to restore the image to its original size for pixel-wise classification.

### 2.3. Backbone Network Branch

The hazards around high-speed railways have complex features in remote sensing images such that they can be confused with the background. Deep learning models often require deeper network layers to accomplish feature extraction. However, deeper networks can increase the cost of training, reduce its efficiency, and lower the capability of generalization of the model to lead to overfitting and network degradation. Therefore, we chose ResNet [20] as the backbone network for downsampling, as shown in Table 1. Residual networks can use the residual structure to construct deep networks and address issues like network degradation and the vanishing gradient. When ResNet is used as the backbone network, layer-wise downsampling can be used to capture rich semantic information that is transmitted to the decoding network.

**Figure 5:**
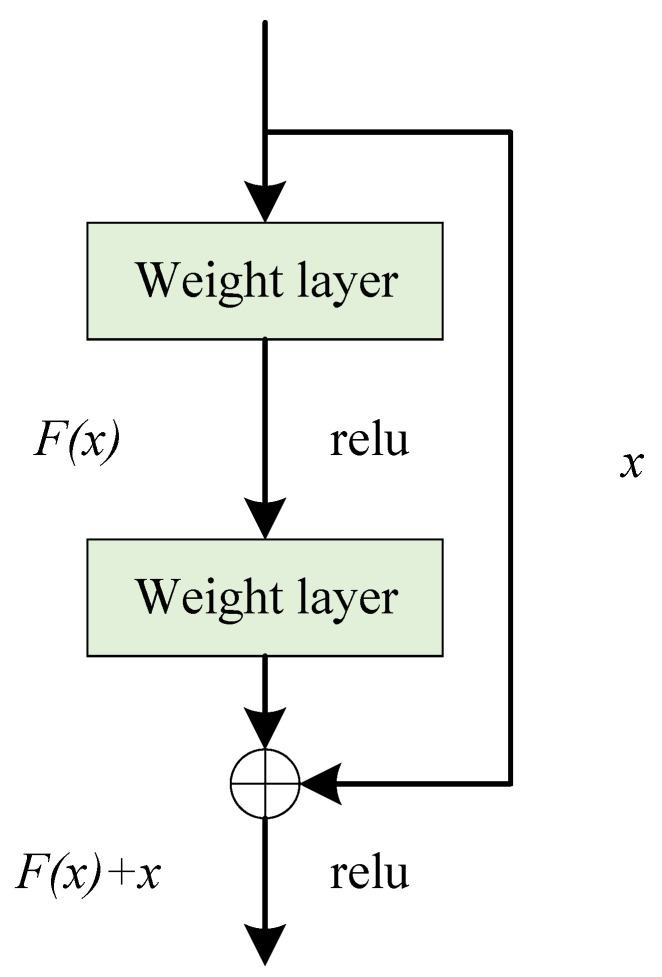
Residual structure.

The residual structure introduced by the residual network can mitigate the degradation in the performance of the model caused by the deepening of the network layers. Its structure is illustrated in Figure 5. The residual unit incorporates a skip connection, because of which the final output function contains an identity term in the process of backward propagation. This solves the problem whereby the gradients become increasingly smaller after several multiplication operations in the deep network. In this way, the error can be propagated back to the shallower layers to significantly enhance the capability of feature extraction of the network.

### 2.4. Multi-Feature Attention Fusion Module (MAFM)

**Figure 6:**
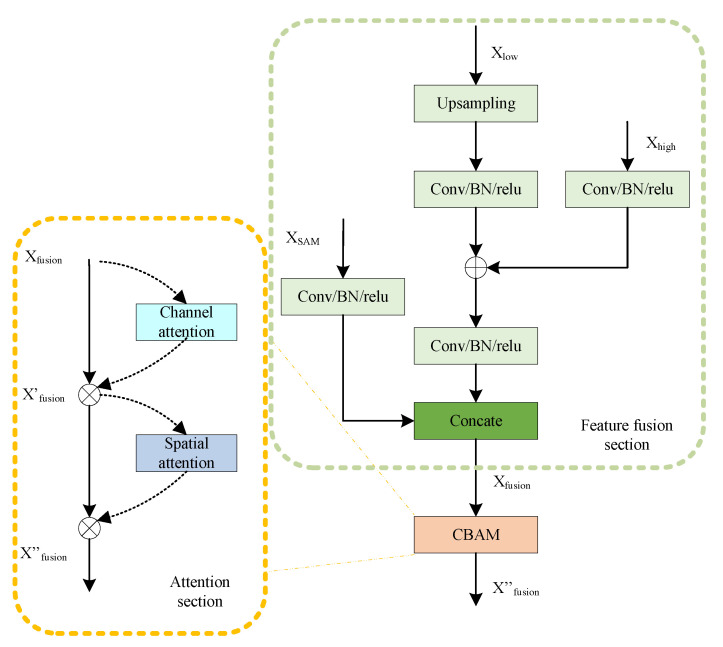
Multi-feature attention fusion module (MAFM).

The main idea of the multi-feature attention fusion module (MAFM) developed here is to reference the skip connections in Unet, integrate contextual information at different scales, and simultaneously fuse the results of segmentation of the SAM. This yields adequate pixel-level attention to the advanced feature maps and enhances the capability of the model to segment hazards of various sizes in images of the environment of high-speed railways. The structure of the module is shown in Figure 6.

The multi-feature attention module consists of three inputs: a low-scale feature map $X_{low}$, a map of features predicted by the SAM $X_{SAM}$, and a high-scale feature map $X_{high}$. The low-scale feature map $X_{low}$ is first subjected to bilinear interpolation during feature fusion, followed by a 3×3 convolution to aggregate information from the channels. This is complemented by the batch normalization and ReLU activation functions to complete the upsampling of the feature map. Weighted addition is subsequently performed on the input feature map predicted by the SAM, and a skip connection is used to concatenate it with the feature map at the same scale in the channel dimension. Equation (1) illustrates the process of feature fusion, where $X_{fusion}$ represents the result of fusion, concat[] represents concatenation in the channel dimension, upsampling() represents bilinear interpolation-based upsampling, and ξ represents the sequential convolution, batch normalization, and ReLU activation operations.

(1) $$X_{fusion}=concat\left[\xi \left(X_{SAM}\right),\xi \left(\xi \left(upsampling\left(X_{low}\right)\right)+\xi \left(X_{high}\right)\right)\right]$$

To enhance the effect of feature fusion, the fusion module introduces a spatial–channel attention mechanism [21] in the self-attention part. This includes both the channel attention and the spatial attention modules. Channel attention aggregates the input image at the spatial scale and infers unique features through average pooling and max pooling. Spatial attention complements channel attention by applying average pooling and max pooling connections along the channel to generate a spatial attention feature map that contains the positions that require attention. The spatial–channel attention process can be represented as follows:

(2) $${X^{\prime }}_{fusion}=M_{c}\left(X_{fusion}\right)⊗X_{fusion}$$

(3) $${X^{\prime \prime }}_{fusion}=M_{s}\left({X^{\prime }}_{fusion}\right)⊗{X^{\prime }}_{fusion}$$

(4) $$M_{c}\left({X^{\prime }}_{fusion}\right)=\delta \left(MLP\left(AvgPool(X_{fusion})\right)+MLP\left(MaxPool(X_{fusion})\right)\right)$$

(5) $$M_{s}\left({X^{\prime }}_{fusion}\right)=\delta \left(f_{7\times 7}\left(concat\left(AvgPool\left(X_{fusion}\right);MaxPool\left(X_{fusion}\right)\right)\right)\right)$$

In the above equations, δ represents the sigmoid function, and AvgPool and Maxpool denote average pooling and max pooling operations, respectively. MLP is a shared-weight two-layer network, $f_{7\times 7}$ represents a convolution operation with a kernel size of 7×7, and ⊗ represents element-wise multiplication. The input image with fused features $X_{fusion}$ sequentially passes through the channel and spatial attention modules, and a feature map updated with attention is finally obtained.

### 2.5. Union Loss Function

The task of extracting targets from remote sensing images often encounters the issue of an unbalanced sample distribution, which is particularly severe when extracting color-coated steel sheet roof buildings in the vicinity of high-speed railways from images. A single deep learning-based loss function considers only a single performance metric, and this often results in training outcomes that do not comprehensively address various parameters. Therefore, we use a union loss function by combining polynomial loss [22] with dice loss [23]. This approach addresses both the problems of sample imbalance and sample similarity to ensure the precision of training of the network. The loss function is expressed as follows:

(6) $$Loss=\lambda Loss_{Poly-N}+\left(1-\lambda \right)Loss_{dice}$$

where $Loss_{Poly-N}$ represents the polynomial loss function, $Loss_{dice}$ represents the dice loss function, and λ is the parameter of weight adjustment. The polynomial loss function decomposes commonly used loss functions (cross-entropy loss and focal loss [24]) into a series of weighted polynomials that can be expressed as follows:

(7) $$Loss_{CE}=-\log\left(P_{t}\right)=\sum_{j=1}^{\infty }1/j{\left(1-P_{t}\right)}^{j}=\left(1-P_{t}\right)+1/2{\left(1-P_{t}\right)}^{2}+\ldots$$

(8) $$Loss_{FL}=-{\left(1-P_{t}\right)}^{\gamma }\log\left(P_{t}\right)={\left(1-P_{t}\right)}^{1+\gamma }+1/2{\left(1-P_{t}\right)}^{2+\gamma }+\ldots$$

In the above equations, $Loss_{CE}$ and $Loss_{FL}$ represent cross-entropy loss and focal loss, respectively, $P_{t}$ is the predicted probability of the target class label, and γ is the modulating factor. Optimizing the loss function by using gradient descent depends on the gradient with respect to $P_{t}$. The derivative of the loss with respect to $P_{t}$ is calculated as follows:

(9) $$-\frac{dLoss_{CE}}{dP_{t}}=\sum_{j=1}^{\infty }{\left(1-P_{t}\right)}^{j-1}=1+\left(1-P_{t}\right)+{\left(1-P_{t}\right)}^{2}+\ldots$$

(10) $$\begin{array}{cc} -\frac{dLoss_{FL}}{dP_{t}} & =\sum_{j=1}^{\infty }\left(1+\gamma /j\right){\left(1-P_{t}\right)}^{j+\gamma -1}\\ \; & =\left(1+\gamma \right){\left(1-P_{t}\right)}^{\gamma }+\left(1+\gamma /2\right){\left(1-P_{t}\right)}^{1+\gamma }+\ldots \end{array}$$

The polynomial terms in gradient expansion have different sensitivities to $P_{t}$. The leading gradient term is one, and it provides a constant gradient that leads to overfitting. The suppression factor γ, introduced by focal loss, caters to scenarios where $P_{t}$ is close to one to avoid overfitting. By following this idea of decomposition, we present polynomial loss as an infinite series of polynomials. In theory, adjusting an infinite number of polynomial coefficients is clearly impractical during training. To address this issue, polynomial loss perturbs the first N important terms in the polynomial coefficients while keeping the remaining ones unchanged. The final polynomial loss function is expressed by considering the first two terms because this is generally sufficient to ensure satisfactory performance.

(11) $$\begin{array}{cc} Loss_{Poly} & =\alpha_{1}\left(1-P_{t}\right)+\alpha_{2}{\left(1-P_{t}\right)}^{2}+\ldots +\alpha_{N}{\left(1-P_{t}\right)}^{N}\\ \; & =\left(\varepsilon_{1}+1\right)\left(1-P_{t}\right)+\ldots +\left(\varepsilon_{N}+1/N\right){\left(1-P_{t}\right)}^{N}+1/\left(N+1\right){\left(1-P_{t}\right)}^{N+1}+\ldots \\ \; & =-\log\left(P_{t}\right)+\sum_{j=1}^{N}\varepsilon_{j}{\left(1-P_{t}\right)}^{j} \end{array}$$

The dice loss function is a metric of set similarity that is commonly used to calculate the similarity between samples. In segmentation tasks, $\left|X\right|$ and $\left|Y\right|$ represent the label of the image and its predicted label, respectively, and “smooth” is the smoothing parameter that is usually set to one to prevent the denominator from being zero.

(12) $$Loss_{dice}=1-\frac{2\left|X⋂Y\right|+smooth}{\left|X\right|+\left|Y\right|+smooth}$$

## 3. Experimental Results and Analysis

### 3.1. Dataset

**Figure 7:**
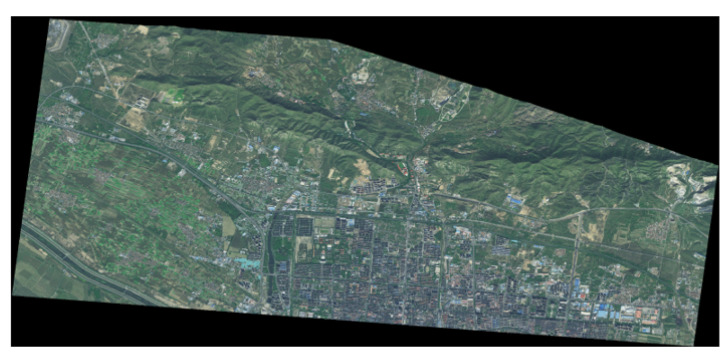
Remote sensing image of the study area.

The datasets used in this study were derived from remote sensing images of the Xuanhua section of the Beijing–Zhangjiakou Railway. The images were captured by the GF-2 satellite in October 2020. The study area is located in Xuanhua District of Zhangjiakou City in Hebei Province, with latitude and longitude coordinates of $40^{\circ }40^{\prime }\;N–40^{\circ }35^{\prime }35\;N$ and $114^{\circ }57^{\prime }\;E–115^{\circ }8^{\prime }\;E$. A remote sensing image of the study area is shown in Figure 7. It has a size of 39,128 × 18,298 and a spatial resolution of 0.8 m.

According to the site survey in Xuanhua District, Zhangjiakou City, the area with a large number of hidden danger targets is selected in the region. Based on the spectral characteristics and shape features of the railway hazardous targets, two railway hazardous datasets were obtained after visual interpretation with the advice of experts in the relevant fields, which were the single-target color-coated steel sheet roof buildings dataset and the multi-target dataset, and the multi-target dataset contained color-coated steel sheet roof buildings, dust nets, and farm mulch.

To validate the effectiveness of the model, we used two publicly available datasets for comparative experiments. The first was the Inria Aerial Image Labeling Dataset (IAIL) [25]. It consisted of aerial orthorectified colored images captured at a resolution of 0.3 m and covering various urban settlements including Chicago, San Francisco, and Vienna, for a total area of 800 ${\mathrm{km}}^{2}$. The second dataset considered here was the Massachusetts Buildings Dataset [26]. It comprised 151 aerial images of the Boston region, each with a resolution of 1500×1500 pixels, and covered an area of approximately 340 ${\mathrm{km}}^{2}$.

### 3.2. Data Pre-Processing and Experimental Parameters

Data pre-processing included label creation, image cropping, image normalization, and data augmentation. Labels in JSON format were converted into common binary image labels during label creation. Due to limitations of the GPU memory of the local hardware and the speed-related requirements of the SAM, the dataset was cropped to a size of 256 × 256 and input to the network. Image normalization increased the speed of convergence of the model. The data augmentation operations included horizontal, vertical, and diagonal flipping as well as the processing of Gaussian noise [27]. The final training dataset is shown in Table 2.

We implemented SAMUnet and the other deep learning models compared with it in PyTorch 2.0.1. Python v3.9 and CUDA version 11.4 were also used. The CPU was Intel i9-10900X (Intel, Santa Clara, CA, USA), and four NVIDIA 3090 GPUs (NVIDIA, Santa Clara, CA, USA) were used as well. Training was conducted for 100 epochs by using the Adam optimizer with a dynamic learning rate. The initial learning rate was set to 0.001, and a cosine strategy for a decay in the rate of learning was used. The batch size for each training epoch was set to 16.

### 3.3. Evaluation Metrics

We used a confusion matrix, which is commonly used in classification tasks, to analyze the classification performed by the models on the test samples and used the results to calculate their parameters. The binary confusion matrix of classification is represented in Table 3. Based on the confusion matrix, we used the following evaluation metrics: precision, recall, F1-score, overall accuracy (OA), and mean intersection over union (IoU) [28,29]. These metrics were calculated as follows:

(13) $$\mathrm{precision}=\frac{\mathrm{TP}}{\mathrm{TP}+\mathrm{FP}}$$

(14) $$\mathrm{recall}=\frac{\mathrm{TP}}{\mathrm{TP}+\mathrm{FN}}$$

(15) $$F1-\mathrm{score}=2\times \frac{\mathrm{precision}\times \mathrm{recall}}{\mathrm{precision}+\mathrm{recall}}$$

(16) $$\mathrm{OA}=\frac{\mathrm{TP}+\mathrm{TN}}{\mathrm{TP}+\mathrm{TN}+\mathrm{FP}+\mathrm{FN}}$$

(17) $$\mathrm{IoU}=\frac{\mathrm{TP}}{\mathrm{TP}+\mathrm{FP}+\mathrm{FN}}$$

Precision represents the ratio of pixels correctly predicted to positive to all pixels predicted to positive, recall represents the ratio of pixels actually undergoing changes to all pixels that are predicted to change, and the F1-score is the harmonic mean of precision and recall. The OA represents the percentage of pixels correctly classified in the image to all pixels, and the IoU is the ratio of the intersection of the union of the true labels to the predicted values in pixel classification.

### 3.4. Comparative Analysis

To confirm the effectiveness of SAMUnet, we compared its performance with that of FCN-8s [30], Segnet [31], Enet [32], Unet [33], PSPnet [34], DeepLabV3+ [35], and Upernet [36]. Table 4 presents the results of these networks on the IAIL and Massachusetts datasets. It is evident that SAMUnet achieved the optimal results on multiple metrics. It outperformed the other networks in terms of precision (1.01% improvement), F1-score (1.08% improvement), OA (0.37% improvement), and IoU (0.7% improvement) on the Massachusetts dataset, with only a slightly lower recall. SAMUnet also delivered superior results on the Aerial dataset, with only a lower precision, but still outperformed the other networks.

**Figure 8:**
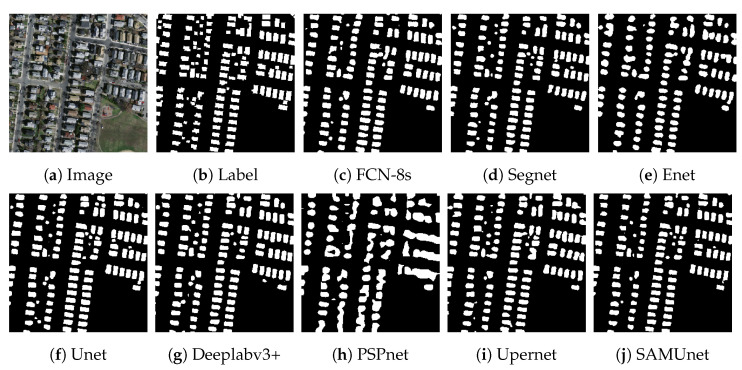
Extraction of small buildings from images in the Massachusetts dataset.

**Figure 9:**
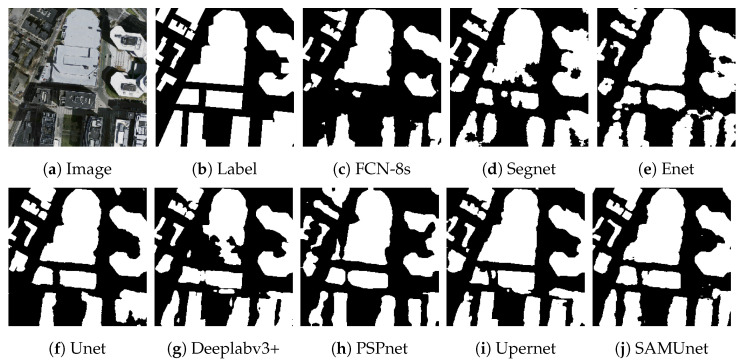
Extraction of large buildings from images in the Massachusetts dataset.

**Figure 10:**
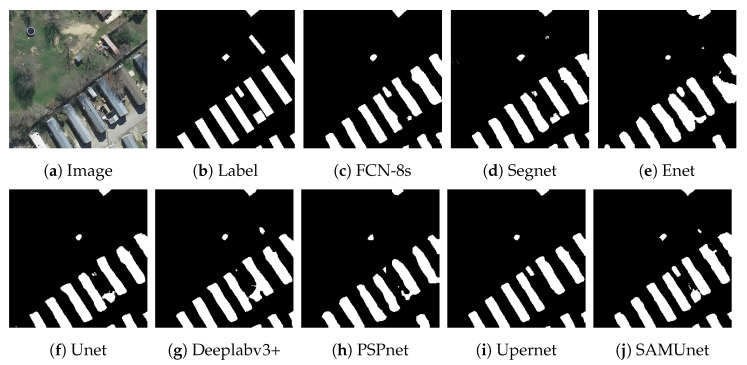
Extraction of small buildings from images in the IAIL dataset.

**Figure 11:**
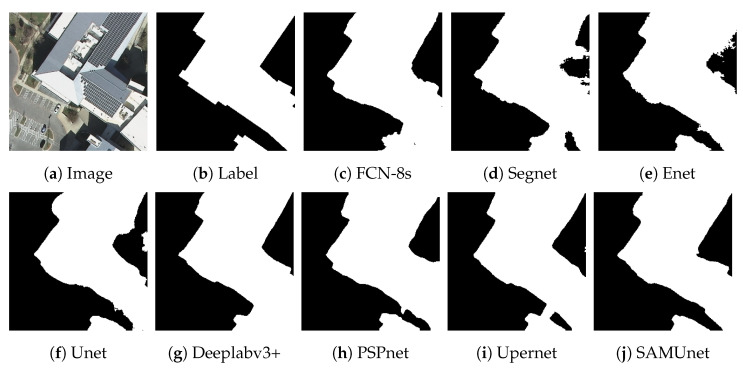
Extraction of large buildings from images in the IAIL dataset.

Figure 8 and Figure 9, respectively, display the results of extraction by all networks on the Massachusetts and the IAIL datasets. SAMUnet exhibited superior capabilities of edge extraction to the other networks and provided smoother and clearer outlines of buildings, especially in densely populated areas with small buildings. Moreover, it demonstrated a better capability of identifying areas with large buildings, particularly in terms of dealing with shadow coverage and identifying the brown roofs of buildings that can be easily confused with the background. Figure 10 and Figure 11 show the results of extraction of buildings from images on the IAIL dataset. SAMUnet exhibited advantages in distinguishing the complex internal structures of large buildings and more precisely extracted the edges of buildings than the other methods.

**Figure 12:**
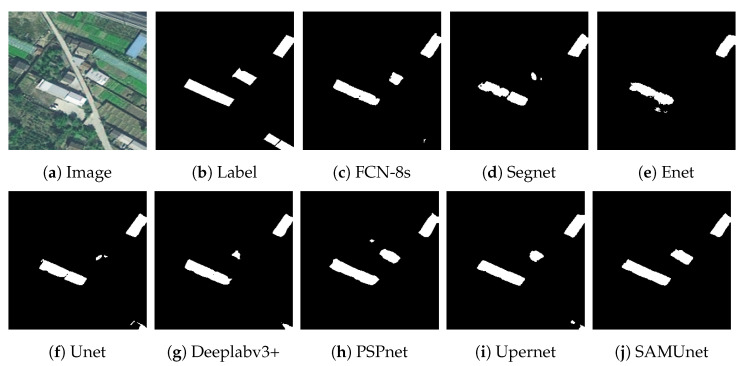
Extraction of small buildings from images in the Railway dataset.

**Figure 13:**
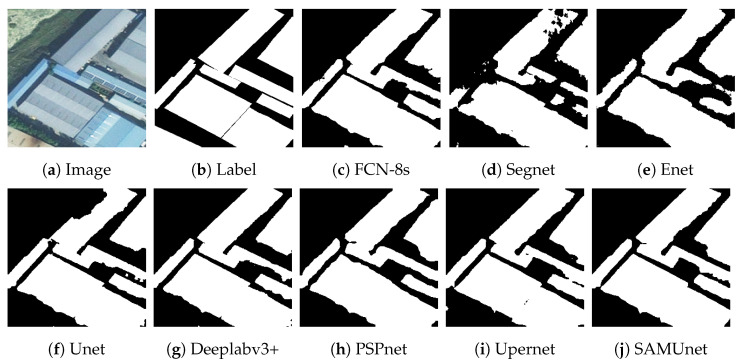
Extraction of large buildings from images in the Railway dataset.

**Figure 14:**
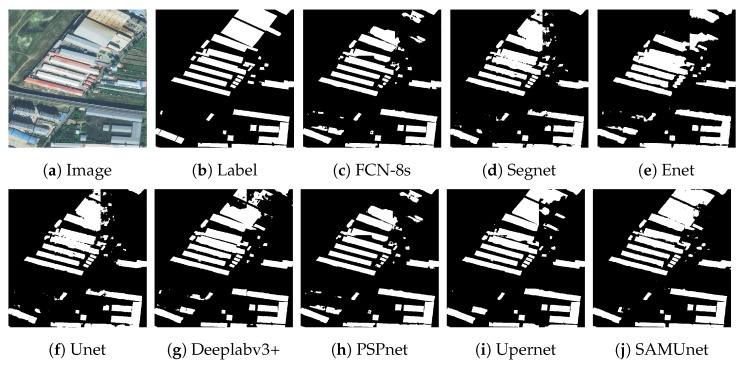
Extraction of large buildings from images in the Railway dataset.

We also applied SAMUnet and the other deep learning models to extract hidden color-coated steel sheet roof buildings in the Xuanhua section of the Jingzhang Railway. The results are presented in Table 5 and Figure 12, Figure 13 and Figure 14. It is clear from them that SAMUnet delivered better performance in extracting both large and small hidden targets than the other methods. While its precision was slightly lower, by 0.01%, than that of Upernet, its performance in terms of the other indicators was significantly better than that of the second-best model, with a recall that was higher by 1.86%, F1-score that was higher 0.72%, OA that was higher by 0.20%, and an IOU by 1.44%. This means that it could accurately extract color-coated steel sheet roof buildings from the images. Its success can be attributed to the feature extraction module of the SAM, which provided pre-classified pixels to compensate for a lack of information on the edges of pixels that the network might have otherwise overlooked. Figure 14 shows the results of extraction of color-coated steel sheet roof buildings from images along a large stretch of the railway. The classical network models were noticeably inaccurate and confused cement surfaces with colored steel roofs and incorrectly identified regular houses as color-coated steel sheet roof buildings. SAMUnet addressed these issues and was able to accurately extract the contours.

### 3.5. Comparative Analysis

SAMUnet uses the SAM module to obtain prior results of segmentation of the target, and uses an attention fusion mechanism to enhance and fuse the features of classification. It filters out useful features to extract hidden hazards in images of the environment of the high-speed railway. To validate the performance of the SAM module and the attention fusion mechanism, we conducted an ablation experiment on the Railway dataset, and the results are shown in Table 6. We used Unet as the baseline, with only its encoding part replaced by ResNet-18 as the base network. The results show that incorporating the SAM branch improved the performance of the model in terms of extracting steel buildings from the images, and all its metrics improved. When all modules were included in the network, its precision improved by 6.28%, recall by 5.09%, OA by 0.87%, and IoU by 4.19% compared with the baseline. This confirms the effectiveness of each module.

### 3.6. Railway Multi-Objective Segmentation Experiment

The actual remote sensing railway hazards segmentation scenario requires segmentation of multiple hazards targets, which have large differences in number, complex spectral features, and serious category imbalance problems. Compared with single-target segmentation, multi-target segmentation places higher requirements on the feature extraction performance of the network, so we conducted experimental comparisons on the Railway multi-target dataset, and the experimental results are as follows Table 7).

**Figure 15:**
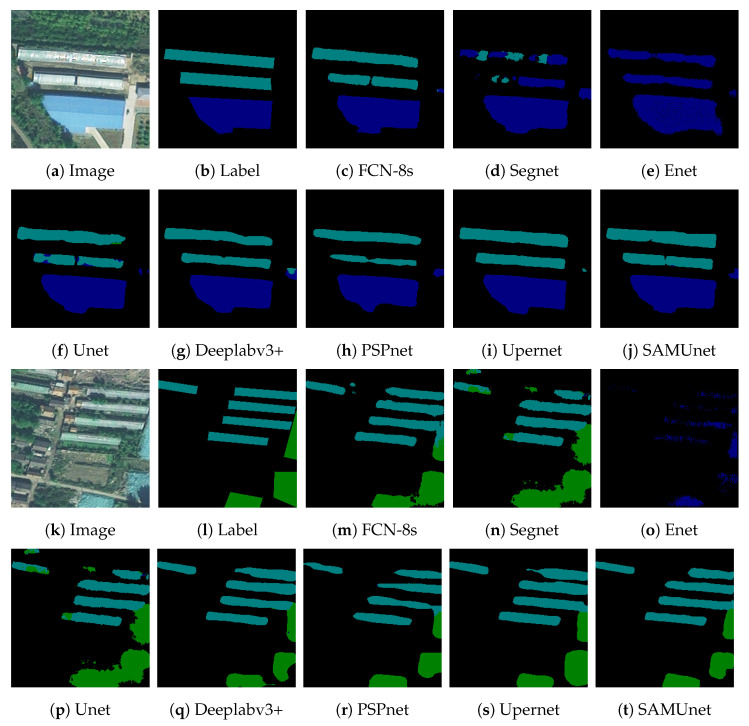
Multi-objective segmentation results for railway hazards.

In the table, CPA represents the PA value for each class, MPA represents the average value of PA for the three classes, CIoU represents the IoU value for each class, and MIoU represents the average value of IoU for the three classes. It can be seen that compared with the traditional network, SAMUnet achieves optimal results in most of the classes, MPA improves by 2.95, and MIoU improves by 1.62. Figure 15 show the segmentation effect of the network; color-coated steel sheet roof buildings are shown in dark blue, farm mulch is shown in light blue and dust nets are shown in green. It can be seen that SAMUnet has a good segmentation result for multi-targets, and it can distinguish between the farm mulch, color-coated steel sheet roof buildings, and dust nets better.

### 3.7. Loss Function and SAM Model Hyperparameter Experiments

In order to reduce the manual interference with the results, the SAM model we used cancels the manual labeling points and uses the automatic labeling mode, the only parameter that can be adjusted is the pre-training model, according to the research content made public by Facebook, the pre-training weights of SAM are vit-b,vit-l,vit-h, the above pre-training model is used to segment the railway hazards, and the segmentation results are showed in Table 8.

**Figure 16:**
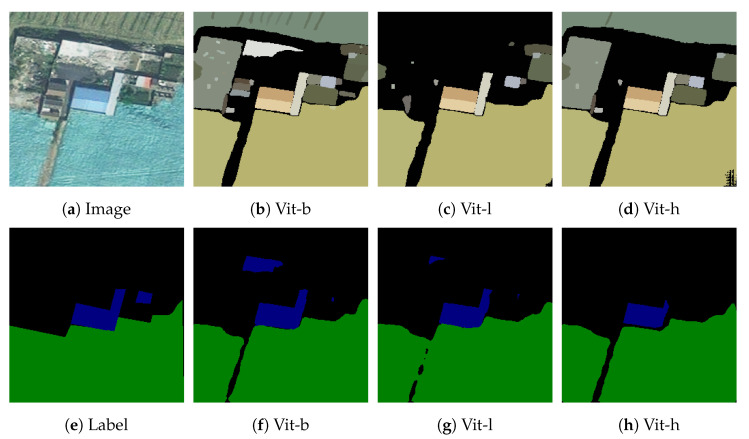
Pre-trained model results under multi-objective railway hazards experiments.

As can be seen in the Table 8, the higher the number of parameters of the pre-training model, the longer the running time, the better the segmentation effect, the difference between the optimal result and the worst result is 0.45% for mpa, and the difference is 1.27% for MIoU. In terms of the running time, there is only a difference of 0.13 s between vit-h and vit-l, but the difference is 1.53 s compared to vit-b, which is close to half of the running time, but the results are not much different. Figure 16 shows the segmentation results of three different pre-training models, and it can be seen that the vit-b model misjudges the background in the middle, while vit-h, vit-l do not have this error, which affects the final segmentation results.

**Figure 17:**
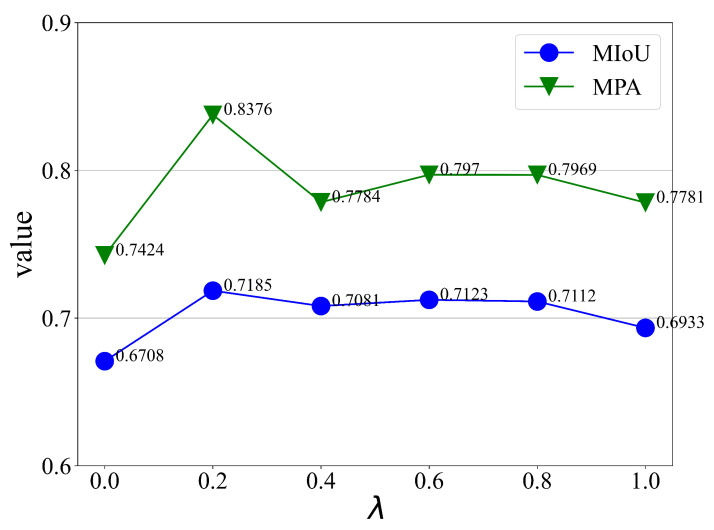
Impact of hyperparameter λ on experiments.

In addition, in order to verify the effect of the union loss function mentioned in Section 2.5 of this paper, we carry out experiments on railway multi-objective segmentation data to verify the effect of the hyperparameter λ value on the experimental results, and the indexes MPA and MIoU obtained from the experiments are shown in Figure 17.

When the hyperparameter λ is taken as 1, the union loss function degrades to Poly loss, and when λ is taken as 0, the union loss function degrades to Dice loss; according to the results, it can be seen that the effect of using separate Dice loss and Poly loss is lower than that of the union loss function, where the optimal result is achieved when λ = 0.2. The reason for this is that Dice loss aims to measure the similarity between the predicted region and the real region, while Poly loss, as a variant of BCE loss and Focal loss, focuses on the categorization of the pixel results and lacks the consideration of the image as a whole, and the union loss function takes into account the advantages of both. In the context of the railway multi-objective dataset with complex background and serious imbalance of the three target samples to be detected, the union loss function can obtain better global inspection effect and local target segmentation accuracy.

## 4. Conclusions

Deep learning-based remote sensing image segmentation of railway hazards can effectively monitor the sources of hazards near the railway, which is of great significance for safeguarding railway operations. Previous studies have targeted the single target of color-coated steel sheet roof buildings, which is not in line with the actual situation, and the effect of traditional feature extraction is limited by the large differences between railway hazards. In this study, the SAMUnet network is proposed to achieve railway multi-target segmentation. The SAM preclassification module combines the large model with the image spectral features to effectively extract the features of railway hazards. The multi-feature fusion module fuses the extracted features using a self-attentive mechanism to enhance the overall capability of the network feature representation.

Experiments and analyses on the public and Railway datasets demonstrate the excellent performance of SAMUnet for railway hazards segmentation, which can simultaneously segment color-coated steel roof buildings, dust nets, farm mulch, and other hazards. Its extraction accuracy outperforms other methods with an overall accuracy of 98.27%, an IoU of 87.9% for single-target railway hazards, MPA of 83.76%, and MIoU of 71.85% for multi-target railway hazards. The results of the ablation experiments validate the effectiveness of each of the modules proposed in this study.e proposed a segmentation network to identify hazards in the environment of high-speed railways that combines the SAM pre-trained large model with the Unet architecture. It is designed to extract and monitor potential threats to the safety of high-speed trains and specifically focuses on hazards such as steel-structured buildings. The SAM module for spectral feature extraction combines the capability of generalization of large models with the spectral features of steel-structured buildings and thus addresses the challenge posed by the difficulty of extraction of complex features from images of areas in which high-speed railways operate. Moreover, the multi-feature fusion module uses self-attention mechanisms to fuse the features of images to enhance the overall capability of feature representation of the network.

## Figures and Tables

**Table 1:** ResNet feature extraction network architecture.

Layers	Output	Structure
Layer 0	112 × 112	7 × 7, 64, 3 × 3 Maxpool
Layer 1	56 × 56	$\left[\begin{array}{c} 3\times 3,64\\ 3\times 3,64 \end{array}\right]\times 2$
Layer 2	28 × 28	$\left[\begin{array}{c} 3\times 3,128\\ 3\times 3,128 \end{array}\right]\times 2$
Layer 3	14 × 14	$\left[\begin{array}{c} 3\times 3,256\\ 3\times 3,256 \end{array}\right]\times 2$
Layer 4	7 × 7	$\left[\begin{array}{c} 3\times 3,512\\ 3\times 3,512 \end{array}\right]\times 2$

**Table 2:** Information on the datasets used for the experiments.

Dataset	Resolution	Number of Images	Area	Image Sizes	Class
Massachusetts	0.3 m	15,225	340 ${\mathrm{km}}^{2}$	256 × 256	Buildings
Aerial	0.3 m	64,980	80 ${\mathrm{km}}^{2}$	256 × 256	Buildings
Railway-CCSS	0.8 m	5045	24 ${\mathrm{km}}^{2}$	256 × 256	CCSS buildings
Railway-multi	0.8 m	1890	24 ${\mathrm{km}}^{2}$	256 × 256	CCSS buildings, dust net, farm mulch

**Table 3:** Binary confusion matrix.

Predicted	Actual	
	**Positive**	**Negative**
**Positive**	TP	FP
**Negative**	FN	TN

**Table 4:** Experimental results on public datasets.

Method	Massachusetts Dataset					IAIL Dataset				
	**Precision**	**Recall**	**F1-Score**	**OA**	**IoU**	**Precision**	**Recall**	**F1-Score**	**OA**	**IoU**
FCN-8s	0.8377	0.6386	0.7284	0.9230	0.7435	0.9136	0.8825	0.8987	0.9772	0.8952
Segnet	0.8330	0.7147	0.7693	0.9307	0.7734	0.8970	0.8819	0.8894	0.9749	0.8865
Enet	0.7896	0.6832	0.7326	0.9193	0.7437	0.8591	0.8240	0.8412	0.9644	0.8433
Unet	0.8241	**0.7684**	0.7953	0.9360	0.7936	0.9125	0.8767	0.8942	0.9763	0.8912
Deeplabev3+	0.8421	0.7499	0.7930	0.9368	0.7929	0.9055	0.8948	0.9001	0.9773	0.8966
PSPnet	0.7901	0.6957	0.7399	0.9209	0.7491	0.8945	0.8830	0.8887	0.9747	0.8858
Upernet	0.8313	0.7615	0.7949	0.9364	0.7936	**0.9146**	0.8872	0.9007	0.9776	0.8972
SAMUnet	**0.8522**	0.7648	**0.8061**	**0.9405**	**0.8037**	0.9072	**0.8981**	**0.9026**	**0.9778**	**0.8989**

Bold represents the optimal value.

**Table 5:** Results of segmentation of color-coated steel sheet roof buildings in images.

Method	Precision	Recall	F1-Score	OA	IoU
FCN-8s	0.8639	0.8178	0.8402	0.9780	0.8505
Segnet	0.8891	0.7264	0.7995	0.9742	0.8476
Enet	0.8343	0.7367	0.7825	0.9710	0.8061
Unet	0.8827	0.7944	0.8363	0.9780	0.8477
DeepLabV3+	0.8848	0.8139	0.8479	0.9793	0.8570
PSPnet	0.8758	0.8367	0.8558	0.9801	0.8634
Upernet	**0.8956**	0.8231	0.8678	0.9807	0.8652
SAMUnet	0.8955	**0.8553**	**0.8750**	**0.9827**	**0.8796**

Bold represents the optimal value.

**Table 6:** Quantitative results of the ablation experiment on the Railway dataset.

Method	Precision	Recall	F1-score	OA	IoU
Baseline	0.8327	0.8044	0.8183	0.9740	0.8377
Baseline + SAM	0.8580	0.8162	0.8366	0.9775	0.8476
Baseline + CBAM	0.8588	0.8196	0.8378	0.9777	0.8493
Baseline + SAM + CBAM	**0.8955**	**0.8553**	**0.8750**	**0.9827**	**0.8796**

Bold represents the optimal value.

**Table 7:** Results of segmentation of railway hazards.

Method	CPA				CIoU				
		**CCSS**	**Dust-Net**	**Farm-Mulch**	**MPA**	**CCSS**	**Dust-Net**	**Farm-Mulch**	**MIoU**
FCN-8s		0.6024	0.6212	0.7551	0.7424	0.5465	0.5706	0.6040	0.6708
Segnet		0.7815	0.6728	0.5893	0.7546	0.5653	0.5482	0.4927	0.6407
Enet		0.6576	-	-	0.4108	0.4755	-	-	0.3567
Unet		0.7734	0.5892	0.7905	0.7837	0.6079	0.5386	0.6245	0.6834
DeepLabV3+		0.7622	0.6443	0.7285	0.7797	0.6127	0.5703	0.6134	0.6902
PSPnet		0.6775	0.6985	0.5550	0.7289	0.5533	0.5861	0.4962	0.6488
Upernet		0.7829	0.6631	0.8037	0.8081	0.6281	0.5913	0.6203	0.7023
SAMUnet		**0.8283**	**0.7345**	**0.8094**	**0.8376**	**0.6374**	**0.5955**	**0.6812**	**0.7185**

Bold represents the optimal value.

**Table 8:** Impact of pre-trained models on experimental results.

Method	Parameters	MPA	MIoU	Running Time
vit-b	86 M	0.8331	0.7058	2.67 s/item
vit-l	307 M	0.8313	0.7151	4.07 s/item
vit-h	632 M	**0.8376**	**0.7185**	4.2 s/item

Bold represents the optimal value.

## Data Availability

The data are not publicly available because the authors do not have permission to share data.
